# Thalidomide-Related Eosinophilic Pneumonia: A case report and brief literature review

**DOI:** 10.1186/1757-1626-1-143

**Published:** 2008-09-08

**Authors:** Lisa Tilluckdharry, Robert Dean, Carol Farver, Muzaffar Ahmad

**Affiliations:** 1 Respiratory Institute, Cleveland Clinic, 9500 Euclid Avenue, Cleveland, Ohio, 44195, USA; 2 Taussig Cancer Institute, Cleveland Clinic, 9500 Euclid Avenue, Cleveland, Ohio, 44195, USA; 3 Pathology and Laboratory Medicine, Cleveland Clinic, 9500 Euclid Avenue, Cleveland, Ohio, 44195, USA

## Abstract

Thalidomide has regained value in the multimodality treatment of leprosy, multiple myeloma, prostate, ovarian and renal cancer. Complications related to arterial and venous complications are well described. However, pulmonary complications remain relatively uncommon. The most common pulmonary side-effect reported is non-specific dyspnea. We report a patient with multiple myeloma, who developed an eosinophilic pneumonia, shortly after starting thalidomide. She had complete resolution of her symptoms and pulmonary infiltrates on discontinuation of the drug and treatment with corticosteroids. Physicians should be cognizant of this potential complication in patients receiving thalidomide who present with dyspnea and pulmonary infiltrates.

## Background

A 68 year-old woman, with multiple myeloma, was admitted with progressive, exertional dyspnea and dry cough, which started a few days before presentation. With exercise, her oxygen saturation fell to 85% on room air. Two weeks prior, she had developed diffuse erythematous skin lesions that spontaneously resolved. She denied fever, chills, rigors, night sweats, weight loss, hemoptysis, myalgias, arthralgias, chest pain, palpitations, orthopnea and paroxysmal nocturnal dyspnea. She had no sick contacts. Two months before, she began treatment with thalidomide 50 mg daily, which was titrated up to 200 mg daily over two weeks. She also received dexamethasone 40 mg for four consecutive days every four weeks. Her past medical history was significant for remote cancers: breast cancer, for which she underwent lumpectomy and radiation; malignant melanoma, which was resected and endometrial cancer, for which hysterectomy and pelvic radiation were performed. There was no previous history of atopy or asthma. On presentation, her medications included aspirin, iron, alendronate, calcium, multivitamin and thalidomide (200 mg daily). She was not on dexamethasone at the time of presentation, but had received her dosing the previous month. She had no drug allergies and never received trimethoprim-sulfamethoxazole prophylaxis. She had no pertinent hobbies, pets, travel history or occupational exposures. Her human immunodeficiency virus status was negative.

On physical examination, she was in mild respiratory distress and on 2 liters of oxygen, her saturation was 96%. Vital signs and cardiac examination were normal. Respiratory examination revealed bibasilar crackles. There were no skin lesions, lymphadenopathy, joint swelling or erythema.

**Figure 1:**
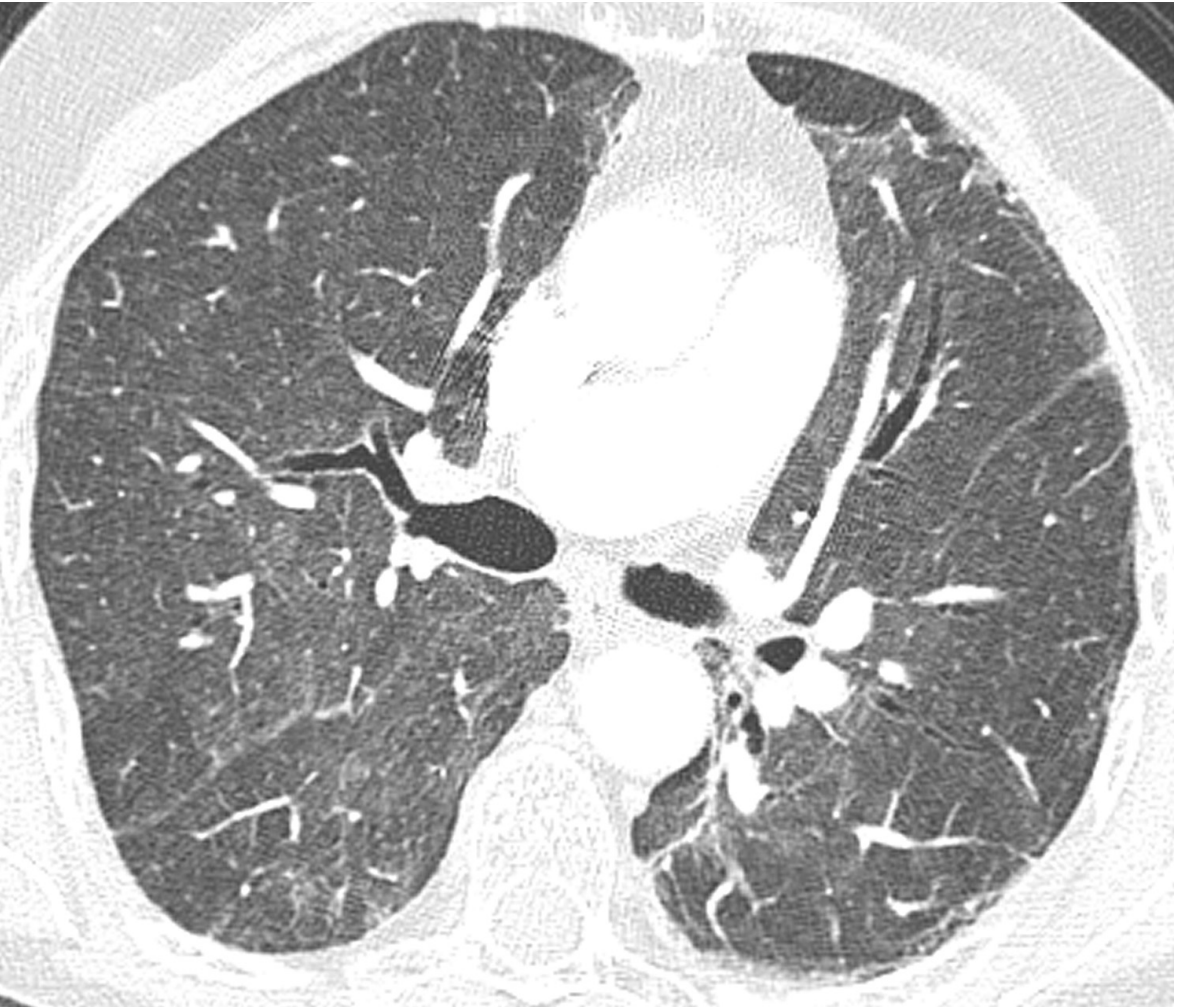
High-resolution transverse CT image shows extensive patchy ground-glass opacity throughout both lungs.

**Figure 2:**
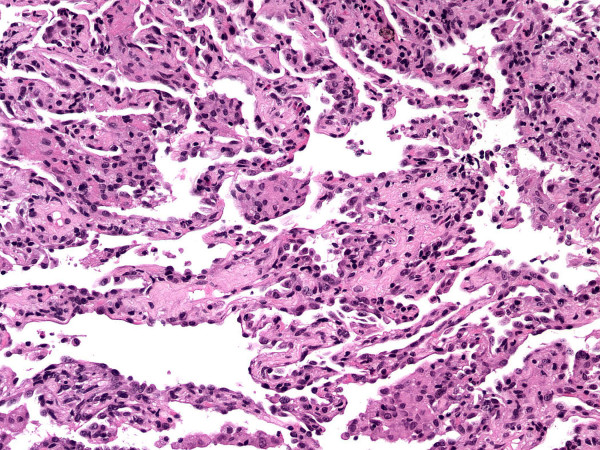
**The lung pathology consists of a mild interstitial pneumonitis with scattered clusters of histiocytes within the alveolar space consistent with loosely-formed granulomas. **(Hematoxylin and eosin, 100×).

On admission, her white blood cell count was 5.6 k/uL (neutrophils 84%, lymphocytes 7.1%, and eosinophils 3%) and her hemoglobin 10.2 g/dl. Her blood chemistries were normal. Computed tomography pulmonary angiography (CTPA) of the chest revealed no pulmonary embolism. There were patchy ground glass infiltrates bilaterally (Fig 1a). Transthoracic echocardiogram revealed normal systolic function and pulmonary pressures. Bronchoscopy was performed to obtain a bronchoalveolar lavage (BAL) specimen and transbronchial biopsies. Specimens from the right middle lobe were sent for cell count and differential, bacterial and fungal cultures, acid fast bacilli stains and cytology. There were 18% lymphocytes and 33% eosinophils on the differential cell count. There was no evidence of cytomegalovirus, bacteria, fungus or Pneumocystis jiroveci. Transbronchial biopsy specimens showed evidence of interstitial pneumonitis with histiocytic clusters suggestive of granulomas (Fig 2). There was no evidence of malignancy. No parasites were seen. These results supported a diagnosis of acute eosinophilic pneumonia.

**Figure 3:**
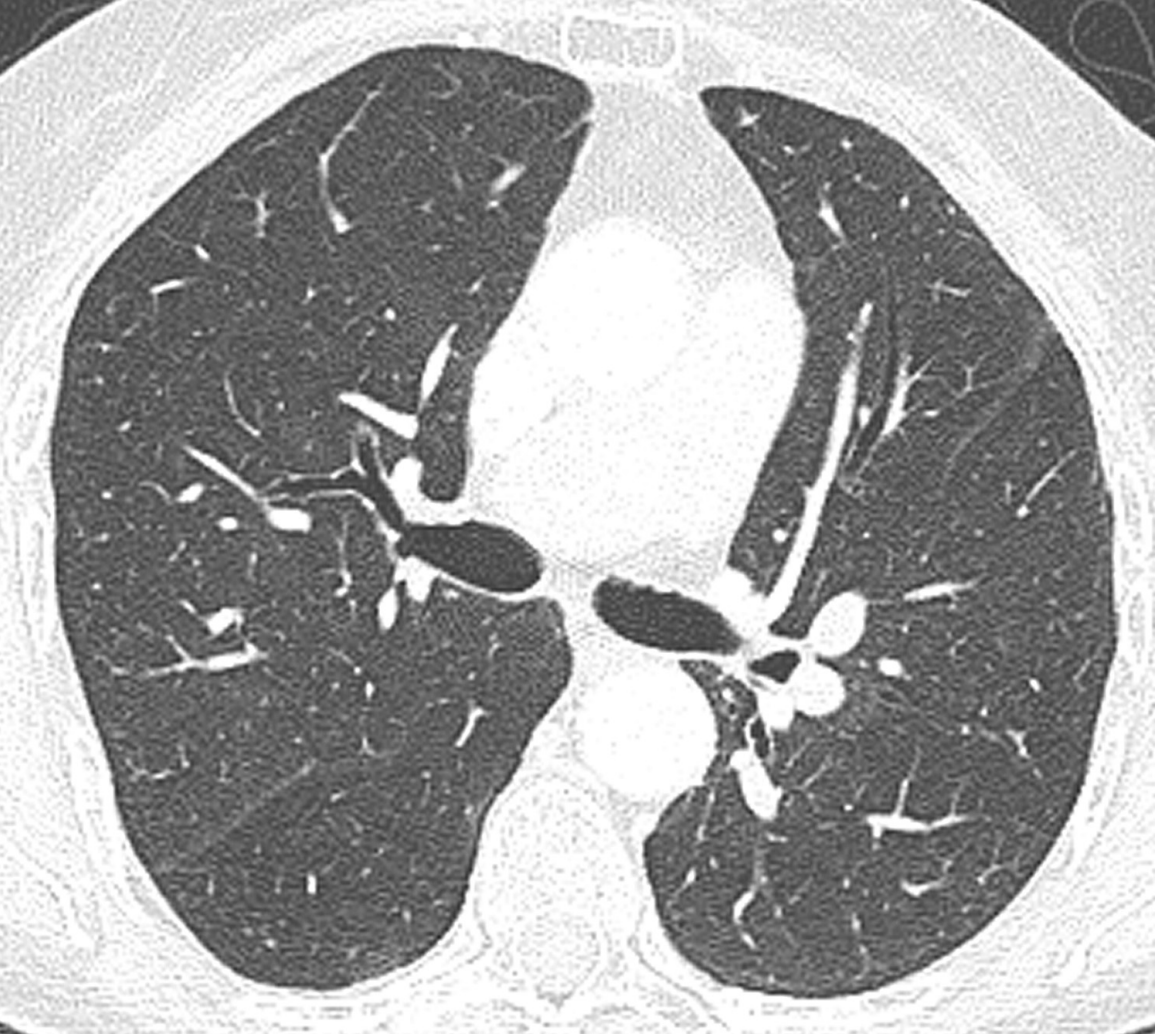
High-resolution transverse CT image at the same levels as figure 1 shows resolution of ground-glass opacity.

The thalidomide was discontinued and she was started on prednisone 40 mg orally daily. She was discharged to home, after three days in the hospital, with minimal dyspnea. At that time, she was saturating 98% on room air, at rest, with no significant desaturation on exercise. Within one week, she was no longer dyspneic. The patient remained symptom free after discontinuation of the steroids. Spirometry and diffusion capacity, one month later, were normal. Repeat CT evaluation showed complete radiographic resolution of the infiltrates (Fig 3).

## Discussion

Thalidomide, originally a sedative-hypnotic drug, has been shown to have activity in the multimodality treatment of leprosy, hematologic malignancies and solid tumors, including prostate, ovarian, renal cell cancer and Kaposi's sarcoma[1,2], due to its antiangiogenic, immunomodulating and anticytokine properties. Angiogenesis, downregulation of tumor necrosis factor, interference with stromal cell/myeloma adhesive properties and stimulation of natural killer cells are thought to be the mechanisms by which it exerts its beneficial effects in multiple myeloma[3].

Serious pulmonary complications due to thalidomide use are not common. The most common pulmonary side-effect is non-specific dyspnea, reported in 4% to 54%[4]. This frequently improves on cessation of the drug. Arterial and venous thromboses, namely, pulmonary embolism and deep vein thromboses, [5-8] are the most serious pulmonary complications and their frequency appears to increase when combined with dexamethasone and other chemotherapeutic drugs [9]. Interstitial pneumonia has been described in case reports in patients with ovarian cancer and multiple myeloma [10-12]. However, none of these patients had significant eosinophilia in the BAL fluid. The case series of three patients with advanced prostate cancer who were treated with a combination of docetaxel and thalidomide, attributed the pulmonary toxicity to the primarily the doxacetal, to which severe interstitial pneumonias had been ascribed[12].

A case report in the Japanese literature, describes a patient with a severe interstitial pneumonia, rash and eosinophilia in the BAL specimen, who required mechanical ventilation. His pneumonia and rash initially responded to discontinuation of the thalidomide and a course of steroids. However, his course was subsequently complicated by methicillin resistant staphylococcus aureus (MRSA) septicemia and he demised [13]. It is not clear from the information provided in the case that the eosinophilia was definitely due to the thalidomide and not an undiagnosed underlying process.

Pleural effusions may occur [14], with the hypersensitivity-type responses [15]. Hypersensitivity pneumonitis has been described with an FDA-approved thalidomide analog, lenalidomide[16].

There is one case report of a patient with multiple myeloma who developed dyspnea and ground glass infiltrates on thalidomide. The bronchoalveolar lavage contained 31% neutrophils, 40% lymphocytes, 20% eosinophils and 1% plasma cells. Transbronchial biopsy specimens revealed fibroblastic plugs in the alveolar spaces with surrounding interstitial inflammation, diagnostic of organizing pneumonia[17].

There are published case reports of patients who developed pulmonary hypertension seemingly due to thalidomide. One case reported described a man with multiple myeloma, who transiently developed severe pulmonary hypertension on thalidomide, which improved on discontinuation and worsened on a rechallenge with the drug[18]. Another case reports a patient with multiple myeloma, who had significant dyspnea, was found to have severe pulmonary hyperstension and subsequently died. There were no other identifiable causes of her pulmonary hypertension on pathology and review of her history and laboratory results[18,19].

To our knowledge, this is the first reported case, in the English literature, of an acute eosinophilic pneumonia, related to thalidomide. The degree of eosinophilia in the BAL fluid, in the absence of any historical, physical or laboratory data to support an alternative cause of eosinophilic pneumonia, strongly supports the diagnosis of an esoinophilic pneumonia. The absence of eosinophils on histology would have been supportive of this diagnosis, but is not required for the diagnosis of eosinophilic pneumonia. Her clinical improvement on discontinuation of the thalidomide suggests that the thalidomide was the most reasonable culprit. This is somewhat confounded by the simultaneous treatment with prednisone, as it can be argued that the patient responded to the steroid, as eosinophilic pneumonias characteristically do and not necessarily to discontinuation of the drug. Resumption of the thalidomide, with recrudescence of the dyspnea and pulmonary infiltrates, would confirm the diagnosis of thalidomide-induced interstitial pneumonia. However, this is not recommended.

## Conclusion

In conclusion, we present a case of eosinophilic pneumonia secondary to thalidomide. The patient's clinical course, radiographic and bronchoscopic findings strongly support a diagnosis of thalidomide-induced eosinophilic pneumonia, in the absence of any other positive historical and laboratory evidence. Physicians should be cognizant of this potential complication in patients receiving thalidomide who present with dyspnea and pulmonary infiltrates.

## Abbreviations

BAL: Bronchoalveolar lavage; CT: Computed Tomography; Ig G: Immunoglobulin G; MRSA: Methicillin-Resistant Staphylococcus Aureus.

## Competing interests

The authors declare that they have no competing interests.

## Authors' contributions

LT managed the patient and analyzed and interpreted the patient data regarding the clinical course of the patient. RD managed the hematological disease and provided follow up. FC performed the pathological examination of the lung biopsy and discussed the features significant to this case. MA was a major contributor in managing the patient and writing the manuscript. All authors read and approved the final manuscript.

## Consent

Written informed consent was obtained from the patient for publication of this case report and accompanying images. A copy of the written consent is available for review by the Editor-in-Chief of this journal.
